# Moderate Laryngeal Dysplasia in the Context of Classification Systems and Clinical Decision Making

**DOI:** 10.1002/lary.32200

**Published:** 2025-04-21

**Authors:** Flurin Mueller‐Diesing, Manuel Stöth, Elena Ludwig, Andreas Rosenwald, Elena Gerhard‐Hartmann, Helena Moratin, Thomas Gehrke, Miguel Goncalves, Matthias Scheich, Stephan Hackenberg, Agmal Scherzad, Till Jasper Meyer

**Affiliations:** ^1^ Department of Oto‐Rhino‐Laryngology, Plastic, Aesthetic and Reconstructive Head and Neck Surgery University of Würzburg Würzburg Germany; ^2^ University of Würzburg, Institute of Pathology Würzburg Germany

**Keywords:** laryngeal dysplasia, laryngeal leukoplakia, premalignant laryngeal lesions, squamous intraepithelial neoplasia, vocal fold leukoplakia

## Abstract

**Objectives:**

Determining the prognosis of vocal fold leukoplakia remains challenging. The 2017 WHO Classification of Head and Neck Tumors proposed a two‐tier grading system for classifying laryngeal precursor lesions. This resulted in the upstaging of “moderate dysplasia” to “high‐grade dysplasia.” The aim of this study was to retrospectively analyze the rates of malignant transformation in relation to the grade of dysplasia.

**Methods:**

Totally, 392 patients who underwent at least one microlaryngoscopy with biopsy for laryngeal leukoplakia were included in this study. The rate and time to malignant transformation were analyzed according to the histopathological diagnosis and the localization of the leukoplakia.

**Results:**

Malignant transformation rates (defined as de novo occurrence of an invasive carcinoma) were low for hyperkeratosis or parakeratosis and mild dysplasia, but comparably high for moderate dysplasia, severe dysplasia, and carcinoma in situ (CIS) (6% vs. 9% vs. 41% vs. 43% vs. 55%). The time between first biopsy and malignant transformation varied widely (0.1–10.8 years) and did not show a significant dependence on the initial histopathological diagnosis.

**Conclusion:**

Based on the observed malignant transformation rates, the data support classifying moderate dysplasia into the “high‐grade” dysplasia group, in line with the fourth WHO classification from 2017. This implies that the clinical management of moderate dysplasia should align with that of severe dysplasia and CIS, involving histologically controlled resection.

**Level of Evidence:**

3.

## Graphical abstract

There is still an ongoing debate about the classification of laryngeal dysplasia. In a large retrospective analysis of 392 patients, the progression of dysplasia and the risk of a de novo occurrence of invasive carcinoma were evaluated. Here especially the high rate of occurrence of invasive carcinoma in the group of moderate dysplasia with 44% is of particular relevance in clinical routine.

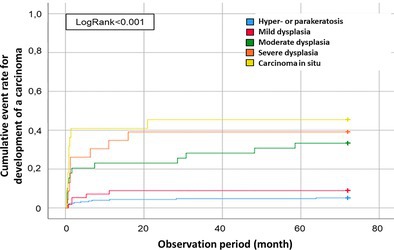

## 1 Introduction

Laryngeal squamous cell carcinoma (LSCC) is the 14th most common malignant tumor in men worldwide and the third most important malignant tumor of the head and neck regardless of gender. Contrary to the trend toward improved 5‐year survival rates for other cancer entities, the 5‐year survival rate for LSCC in the USA fell from 66% to 61% between 1975 and 2019 [1]. The location of the tumor is of great clinical and prognostic relevance. A distinction is made between subglottic, glottic, and supraglottic LSCC. With up to 69%, glottic LSCC makes up the largest proportion in Germany [2]. Glottic LSCC in earlier tumor stages (T1, T2, N0) can usually be treated larynx‐preserving with a satisfactory 5‐year survival rate of 82%–100% [3, 4]. In advanced tumor stages, survival rates are significantly poorer, and the therapy is associated with a significantly greater loss of quality of life [5]. In view of this, early diagnosis and prevention play a decisive role. In addition to abstinence from harmful substances, another key factor is the early detection, sufficient treatment, and close follow‐up of precursor lesions [6, 7].

Precursor lesions usually present as so‐called leukoplakia, a white mucosal lesion of the vocal folds, less frequently also as erythematous mucosal lesions (erythroplakia) or papillomatous changes of the vocal folds. In 1978, the WHO defined leukoplakia as “A white patch or plaque that cannot be characterized clinically or pathologically as any other disease.” [8] This implies that the risk of malignant transformation and, in many cases, the nature of individual lesions cannot be determined solely by endoscopic investigations. Therefore, bioptic evaluation and histopathological assessment are crucial for guiding further clinical management.

Based on the histological changes, there are different levels of disruption of the epithelial morphology, which determine the grade of squamous intraepithelial lesions. These include altered epithelial stratification, abnormal maturation, and an increase in the number of mitoses as well as in the more severe forms superficial mitoses. Furthermore, the presence and extent of cytological atypia are important for the histopathological classification of such lesions, including abnormal variation in nuclear size and shape. Given the variety of histological changes, it is not surprising that the histopathological classification of laryngeal precursor lesions in practice remains to some extent subjective. To address these challenges, several classification systems have been developed over the past six decades with the aim of more accurately quantifying and categorizing laryngeal precursor lesions [9, 10, 11]. Between 1978 and 2005, the WHO provided three classifications that distinguish between mild, moderate, and severe dysplasia, and carcinoma in situ (CIS) [9]. Other frequently used classifications are the Ljubljana classification, which categorizes laryngeal squamous lesions into three hyperplasia grades in addition to CIS, and the squamous intraepithelial neoplasia (SIN) grading, which splits into SIN grades I–III, with both high‐grade dysplasia and CIS summarized under grade III SIN [9]. None of the latter three classifications proved to be superior, especially with regard to interobserver variability [12, 13]. Fleskens' study showed that none of the three classifications exceeded a *κ* value of 0.55 [12, 13]. To reduce investigator dependence, a fourth new WHO classification simplified the degrees of the lesions into two categories: “low‐grade” and “high‐grade” dysplasia, with an optional differentiation of CIS from “high‐grade” dysplasia [9, 10]. In this classification, moderate dysplastic lesions were assigned to the “high‐grade” dysplasia group. The newly introduced two‐tiered or respectively three‐tiered system was also continued in the current 5th WHO classification [14].

The different classification systems and the high intraobserver and interobserver variability of histopathological assessment often result in a very heterogeneous picture regarding the clinical significance of individual dysplasia grades.

This is further complicated by the fact that studies over a long observation period are necessary to evaluate the transformation risk over the years. For example, Weller et al. described an average transformation rate of 5.8 years [15].

To avoid undertreatment or overtreatment, it is essential to find a classification system that determines the risk of malignant transformation with the highest possible accuracy.

The aim of the presented study is to retrospectively evaluate the risk of malignant transformation in accordance with the histopathological findings of the initial glottic leukoplakia biopsy.

## 2 Materials and Methods

### 2.1 Data Collection and Study Cohort

This study was approved by the institutional ethics committee on human research of the Julius‐Maximilians‐University Würzburg (reference number 20200306 01).

The charts of all patients, who underwent a microlaryngoscopy with a vocal fold biopsy between January 2003 and September 2018, were screened. All patients with laryngeal fold leukoplakia and the histopathological diagnosis of hyperkeratosis or parakeratosis, mild, moderate, or severe dysplasia, or CIS at first microlaryngoscopy were included in this study. Exclusion criteria were histopathological diagnosis of invasive LSCC in the initial biopsy, previous laryngeal surgery, or laryngeal malignancies in the medical history.

**FIGURE 1:**
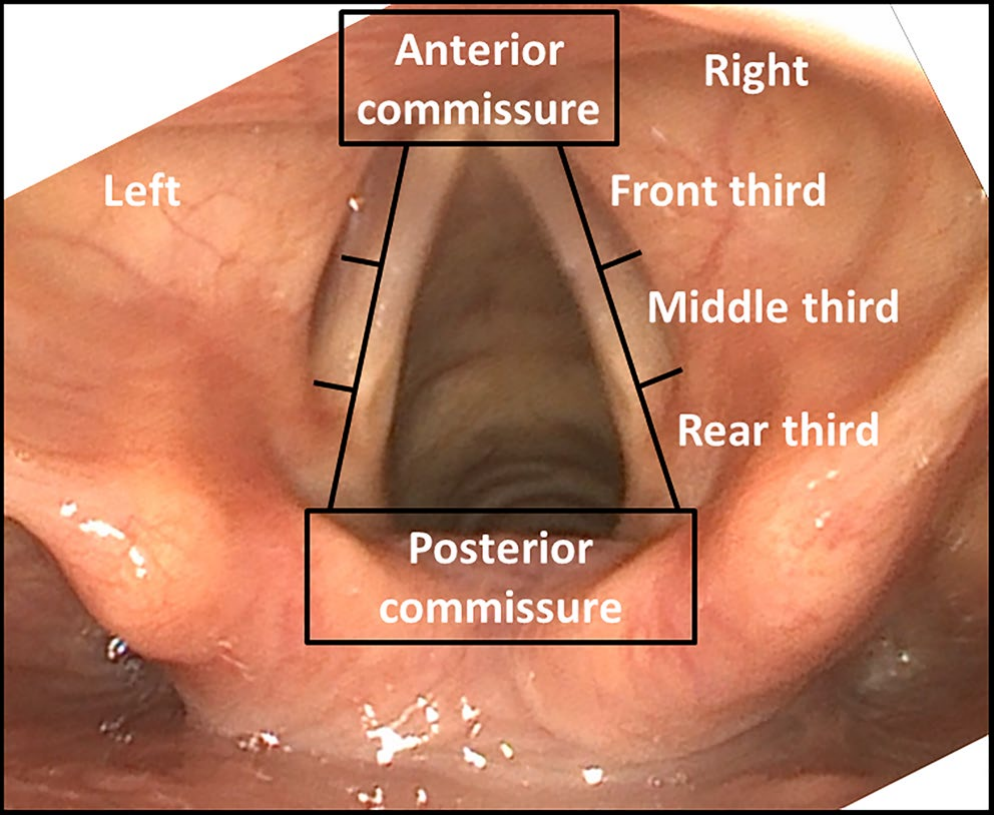
Partitioning of the larynx for describing the localizations of the leukoplakias: Anterior commissure, vocal fold front third right, vocal fold front third left, vocal fold middle third right, vocal fold middle third left, vocal fold rear third right, vocal fold rear third left, and posterior commissure.

The following data were retrieved from electronic medical records: For the patients baseline characteristics, age, gender, consumption of noxious substances, localization of the laryngeal leukoplakia based on the operative report, histological diagnosis of the initial and all follow‐up laryngeal biopsies, and the time from the first microlaryngoscopy to all subsequent follow‐up microlaryngoscopies were recorded. If more than one histology was taken within one surgery, the histology with the higher grade was registered. The localizations of vocal fold leukoplakia were categorized into 10 areas (see Figure 1), by reviewing the operative reports.

According to the local institutional therapy concept—all patients with a final histopathological diagnosis of a severe laryngeal dysplasia or CIS underwent a follow‐up microlaryngoscopy with a complete resection and histology‐controlled margins. In all other cases, patients underwent periodic clinical follow‐up examinations, including flexible fiberoptic laryngoscopy. In cases of new leukoplakia lesions, as well as in cases of an increase in size or change in the surface characteristics of known leukoplakia, a microlaryngoscopy with biopsy was subsequently performed.

The incidence of the different histological findings at the various localizations, the influence of noxious exposure on the risk of carcinoma development, and the transformation rate to a lesion of higher grade, including malignant transformation of the different histologies at initial diagnosis, were analyzed. Malignant transformation was defined as de novo occurrence of an invasive carcinoma.

### 2.2 Data Interpretation and Statistical Analysis

All data were transferred to standard spreadsheets. The statistical analysis was performed by SPSS Statistics Version 26.0.0.0 (IBM, Armonk, NY, USA). For normally distributed data, the values were compared using the *t*‐test for independent samples. An ANOVA was used to compare the values of more than two groups.

When comparing nominal or ordinal‐scaled data, a cross table was used, followed by a chi‐square test. Unless otherwise stated, all data were presented as mean ± standard deviation. A *p* value < 0.05 was set as statistically significant.

## 3 Results

### 3.1 Patient Characteristics

In the period from January 1, 2003 to December 31, 2017, a total of 392 patients with laryngeal precursor lesions underwent at least one surgical procedure in our clinic.

The age of the patients with a diagnosis of low‐grade dysplasia was compared with the group of patients with high‐grade dysplasia. On average, the group with low‐grade dysplasia was significantly younger at the time of initial diagnosis, with an average age of 57.2 years, compared to the group with high‐grade dysplasia, which had an average age of 66.8 years (*p* < 0.001).

The mean follow‐up was 2.7 years (± 3.21 years), calculated by the time between the date of the first surgery and the date of the last appointment in the outpatient department.

### 3.2 Localization and Histopathological Findings

A total of 1048 leukoplakic lesions at various locations of the larynx were described in the initial microlaryngoscopy report of the 392 patients. The locations were categorized into eight areas (see Figure 1) and the incidence of leukoplakia in the individual regions was evaluated. The most frequent locations of leukoplakia were the anterior third of the vocal cord, with a total of 41% of the cases (see Table 1).

**TABLE 1:** Rate of malignant transformation in relation to the leukoplakia localizations in the first microlaryngoscopy (*n* = 1048 leukoplakia locations in 392 patients): High progression rates to invasive carcinoma were seen for leukoplakia localizations at the anterior commissure (25.5%), moderate at the vocal fold front third right and left (mean 15.9%), vocal fold middle third right and left (mean 15.4%), vocal fold rear third right and left (mean 20.8%), and low at the posterior commissure (2.5%).

Glottic leukoplakia localization	Number of leukoplakia sites, *n* (%)	Progression to malignancy, *n* (%)
Anterior commissure	51 (4.9)	13 (25.5)
Vocal fold front third right	222 (21.2)	34 (15.3)
Vocal fold front third left	207 (19.8)	34 (16.4)
Vocal fold middle third right	165 (15.7)	28 (17.0)
Vocal fold middle third left	166 (15.8)	23 (13.9)
Vocal fold rear third right	103 (9.8)	22 (21.4)
Vocal fold rear third left	94 (9.0)	19 (20.2)
Posterior commissure	40 (3.8)	1 (2.5)
Sum *n* (%)	1048 (100)	174 (100)

In 40.1% of cases, bilateral lesions were detected during initial microlaryngoscopy. There was no significant difference in the risk of malignant transformation in unilateral leukoplakia compared to bilateral leukoplakia (15.3% vs. 13.4%; *p* = 0.59) (see Table 2). However, when considering all 1048 described leukoplakia localizations, it became noticeable that a lower proportion of the severe dysplasia and CIS was localized in the region of the anterior and middle thirds of the vocal fold than the leukoplakia without dysplasia and the mild and moderate dysplasia (see Table 3). In contrast, there was a higher proportion of severe dysplasia and CIS compared to leukoplakia without dysplasia localized in the anterior commissure (see Table 3).

**TABLE 2:** Correlation of clinical factors in relation to the histology of the initial laryngeal biopsy (*n* = 392). Depending on the histology of the first biopsy taken, there was a significant difference in age and the number of all laryngoscopies.

Clinical factors	Frequency *n* (%) or mean (± SD)	Hyperkeratosis or parakeratosis *n* (%)	Mild dysplasia *n* (%)	Moderate dysplasia *n* (%)	Severe dysplasia *n* (%)	Carcinoma in situ *n* (%)	*p*
Gender							
Male	314 (80.1%)	195 (77.4%)	43 (76.8%)	33 (84.6%)	22 (95.7%)	21 (95.5%)	0.1069
Female	78 (19.9%)	57 (22.6%)	13 (23.2%)	6 (15.4%)	1 (4.3%)	1 (4.5%)	—
Age							
Years	59.3 (± 11.7)	57.0 (± 11.7)	57.9 (± 11.1)	66.08 (± 9.2)	66.5 (± 9.1)	68.2 (± 9.3)	< 0.001
Smoking							
Ever smoker	246 (85.1%)	158 (83.6%)	36 (92.3%)	22 (81.5%)	18 (90%)	12 (85.7%)	0.1378
No	43 (14.9%)	31 (16.4%)	3 (7.7%)	5 (18.5%)	2 (10%)	2 (14.3%)	—
Site							
Unilateral disease	235 (59.9%)	152 (60.3%)	30 (53.6%)	26 (66.7%)	16 (69.7%)	11 (50%)	0.1001
Bilateral disease	157 (40.1%)	100 (39.7%)	26 (46.4%)	13 (33.3%)	7 (30.3%)	11 (50%)	—
Number of all laryngoscopies							
*n* (± average)	392	1.7 (± 1.8)	2.3 (± 2.4)	3.6 (± 3.8)	4.9 (±3.3)	4.1 (± 3.1)	< 0.001
Time progression to invasive carcinoma							
Months	392	13.6 (± 25.2, median: 2.5)	3.4 (± 3.6, median: 1.4)	27.3 (± 39.2, median: 3.8)	10.5 (± 22.1, median: 1.0)	15.4 (± 31.6, median: 0.7)	0.4729

**TABLE 3:** Glottic leukoplakia histology (in case of multiple biopsies, the highest histology grade was considered) in dependence on the localization of the clinically described leukoplakia localizations in the first microlaryngoscopy (*n* = 1048 leukoplakia lesions in 392 patients): In sum, 4.9% of the described leukoplakia were observed at the anterior commissure, 40.9% at the front third of the vocal fold on both sides, 31.6% at the middle third of the vocal fold on both sides, 18.8% at the rear third of the vocal fold on both sides, and 3.8% at the posterior commissure.

Glottic leukoplakia localization	Hyperkeratosis or parakeratosis, *n* (%)	Mild dysplasia, *n* (%)	Moderate dysplasia, *n* (%)	Severe dysplasia, *n* (%)	Carcinoma in situ, *n* (%)	Total, *n* (%)
Anterior commissure	23 (3.6)	8 (4.7)	7 (6.9)	6 (8.8)	7 (9.3)	51 (4.9)
Vocal fold front third right	134 (21.1)	38 (22.5)	24 (23.5)	12 (17.6)	14 (18.7)	222 (21.2)
Vocal fold front third left	134 (21.1)	27 (16.0)	18 (17.6)	12 (17.6)	16 (21.3)	207 (19.8)
Vocal fold middle third right	94 (14.8)	33 (19.5)	19 (18.6)	9 (13.2)	10 (13.3)	165 (15.7)
Vocal fold middle third left	104 (16.4)	26 (15.4)	12 (11.8)	12 (17.6)	12 (16.0)	166 (15.8)
Vocal fold rear third right	58 (9.1)	18 (10.7)	13 (12.7)	6 (8.8)	8 (10.7)	103 (9.8)
Vocal fold rear third left	56 (8.8)	13 (7.7)	8 (7.8)	9 (13.2)	8 (10.7)	94 (9.0)
Posterior commissure	31 (4.9)	6 (3.6)	1 (1.0)	2 (3.0)	0 (0.0)	40 (3.8)
Sum *n* (%)	634 (60.5)	169 (16.1)	102 (9.7)	68 (6.5)	75 (7.2)	1048 (100)

In the following evaluation of the histopathological results, for each patient only the biopsy with the highest grade of dysplasia was included, leaving in total 392 lesions. The largest fraction of 252 patients showed hyperplasia or parakeratosis without dysplasia, 56 patients had mild dysplasia, 39 patients had moderate dysplasia, 23 patients had severe dysplasia, and 22 patients had CIS (64.3%, 14.3%, 9.9%, 5.9%, 5.6%) (see Tables 3 and 4).

**TABLE 4:** Rate of malignant transformation in relation to the histology of the initial laryngeal biopsy (*n* = 392 patients): Low progression rates to malignancy were observed for hyperkeratosis or parakeratosis (6%) and mild dysplasia (9%), high progression rates for moderate dysplasia (41%), severe dysplasia (43%) and carcinoma in situ (55%). Calculating “low‐grade” dysplasia and “high‐grade” dysplasia models result in a low (6%) and a high (45%) progression rate.

Initial histopathological diagnosis	Number of cases, *n* (%)	Progression to invasive carcinoma, *n* (%)
Hyperkeratosis or parakeratosis	252 (64)	14 (6)
Mild dysplasia	56 (14)	5 (9)
“Low‐grade” dysplasia (WHO 2017)	308 (79)	19 (6)
Moderate dysplasia	39 (10)	16 (41)
Severe dysplasia	23 (6)	10 (43)
Carcinoma in situ	22 (6)	12 (55)
“High‐grade” dysplasia (WHO 2017)	84 (21)	38 (45)

### 3.3 Histology Over the Course of Time

A total of 77 (19.6%) patients showed progression to a lesion of higher grade within a follow‐up biopsy.

Of 252 patients with an initial diagnosis of hyperkeratosis or parakeratosis without dysplasia, four patients developed mild dysplasia, four patients developed moderate dysplasia, and one patient developed severe dysplasia (1.6%, 1.6%, 0.4%). Three patients were diagnosed with CIS and 14 patients with squamous cell carcinoma (1.2%, 5.6%). The majority of patients (89.7%) showed no de novo occurrence of dysplasia.

In the group of patients with initially mild dysplasia, a total of nine (16.1%) patients progressed to a lesion of higher grade. Of these, two patients (3.6%) developed moderate grade dysplasia and a further two (3.6%) developed severe grade dysplasia. Five (8.9%) patients were later diagnosed with laryngeal carcinoma.

Out of 39 patients with initially moderate dysplasia, one (2.6%) patient developed severe dysplasia, and 16 (41%) patients developed invasive squamous cell carcinoma in follow‐up.

The subsequent histopathological findings of patients who initially presented with severe dysplasia showed CIS in three (13%) patients and squamous cell carcinoma in 10 (43.5%) patients.

Of 22 patients who had previously been diagnosed with CIS, 12 (54.5%) developed invasive laryngeal carcinoma during the observation period.

Looking at the rate of transformation into laryngeal carcinoma, a total of 57 patients (14.5%) over all groups developed an invasive carcinoma during the observation period.

There was no significant difference in malignant transformation risk between patients with leukoplakia with no dysplasia (6%) and those with mild dysplasia (9%) (*p* = 0.34). However, patients with moderate dysplasia had a significantly higher risk of progression to invasive carcinoma compared to both groups. Specifically, 41% of patients with moderate dysplasia developed carcinoma versus 6% with no dysplasia (*p* < 0.001) and 9% with mild dysplasia (*p* = 0.03). Similarly, patients with severe dysplasia had a significantly higher risk compared to those with mild dysplasia (43% vs. 9%, *p* = 0.017). Interestingly, the transformation risk of patients with moderate dysplasia was comparable to those with severe dysplasia and carcinomas in situ (41% vs. 43% vs. 55%).

The malignant transformation time in leukoplakia without dysplasia was on average 13.6 (± 25.2) months, 3.4 (± 3.6) months for mild dysplasia, 27.3 (± 39.2) months for moderate dysplasia, 10.5 (± 22.1) months for severe dysplasia, and 15.4 (± 31.6) months for CIS. There were no significant differences in the time to malignant transformation between the groups.

If the histopathological grade of dysplasia is retrospectively grouped into the new binary categories “low grade” or “high grade” as per the 2017 WHO classification, with the moderate dysplasias classified as “high grade,” the following trends emerge. During the observation period, 6% of “low‐grade” and 45% of “high‐grade” dysplasias progressed to invasive squamous cell carcinoma. Thus, there was a significantly higher rate of malignant transformation from “high‐grade” dysplasias (*p* < 0.001). With regard to the time from the initial diagnosis of a laryngeal precursor lesion to the diagnosis of laryngeal carcinoma, the range of time for malignant progression was 1–130 months (0.1–10.8 years).

## 4 Discussion

To determine the appropriate treatment extent for a patient with laryngeal leukoplakia, it is essential for the treating physician to understand the patient's individual prognosis. Overtreatment of vocal fold leukoplakia can lead to severe impairment of voice function, while under treatment with inadequate resection margins leads to a high risk of recurrence and progression to cancer. The risk of malignant transformation is strongly associated with the grade of dysplasia [9, 16, 17]. There is a scientific and clinical consensus about the high risk of malignant transformation for severe dysplasia and CIS. The histopathological diagnosis of severe dysplasia or CIS in the biopsy implies a second microlaryngoscopy with histology‐controlled leukoplakia resection. However, there is an ongoing discussion about the clinical consequence in the case of moderate dysplasia [17]. In the study presented here, a high malignant transformation rate of moderate dysplasia (41%) comparable to those of severe dysplasia (44%) and CIS (55%) was observed. Other studies observed heterogeneous malignant transformation rates of moderate dysplasia diagnosed in the first biopsy, ranging from 0% [18, 19] to 48% [20] (see literature review Table 5). However, the criteria for histopathological classification have been inhomogeneous, making it difficult to compare results across studies. In addition, the interobserver variation is highest in the definition of moderate dysplasia [32].

**TABLE 5:** Literature review of published progression rates depending on the first histopathology diagnosis.

Study	Hyperkeratosis or parakeratosis, *n* cancer/*n* (%)	Mild dysplasia, *n* cancer/*n* (%)	Moderate dysplasia, *n* cancer/*n* (%)	Severe dysplasia, *n* cancer/*n* (%)	Carcinoma in situ, *n* cancer/*n* (%)	Sum, *n* cancer/*n* (%)
Present study	14/252 (5.6)	5/56 (8.9)	16/39 (41.0)	10/23 (43.5)	12/22 (54.5)	57/392 (14.5)
Karatayli‐Ozgursoy [21]	—	1/17 (5.9)	2/13 (15.4)	1/25 (4)	5/52 (9.6)	9/107 (8.4)
Luers [20]	—	7/26 (26.9)	12/25 (48)	8/19 (42.1)	—	27/70 (38.6)
Theodosiou [22]	—	10/24 (41.7)	8/18 (44.4)	11/17 (64.7)		29/59 (49.2)
Rohde [23]	—	1/18 (5.6)	1/16 (6.3)	7/35 (20)	6/32 (18.8)	15/101 (14.9)
Zhang [24]	—	0/22 (0)	5/25 (20)	2/14 (14.3)	10/25 (40)	17/86 (19.8)
Spielmann [25]	—	7/30 (23.3)		8/15 (53.3)		15/45 (33.3)
Jeannon [26]	—	1/23 (4.1)	13/64 (20.3)	14/26 (53.8)	—	28/114 (24.6)
Ricci [27]	2/86 (2.3)	2/42 (4.8)	5/36 (13.9)	3/21 (14.3)		12/185 (6.5)
Gallo [28]	6/143 (4.1)	4/56 (7.1)	6/28 (21.4)	3/32 (9.3)		19/259 (7.3)
Plch [18]	0/24 (0)	0/63 (0)	0/25 (0)	3/7 (42.9)	3/4 (75)	6/123 (4.9)
Uno [19]	2/14 (14.3)	2/10 (20)	0/9 (0)	2/7 (28.6)	—	6/40 (15)
Blackwell [29]	—	3/26 (11.5)	5/15 (33.3)	4/9 (44.4)	1/9 (11.1)	13/59 (22)
Højslet [30]	—	6/151 (4)	4/9 (44.4)	4/10 (40)		14/170 (8.2)
Sllamniku [31]	18/604 (3)	15/204 (7.4)	4/23 (17.4)	25/90 (27.8)	—	62/921 (6.7)

**FIGURE 2:**
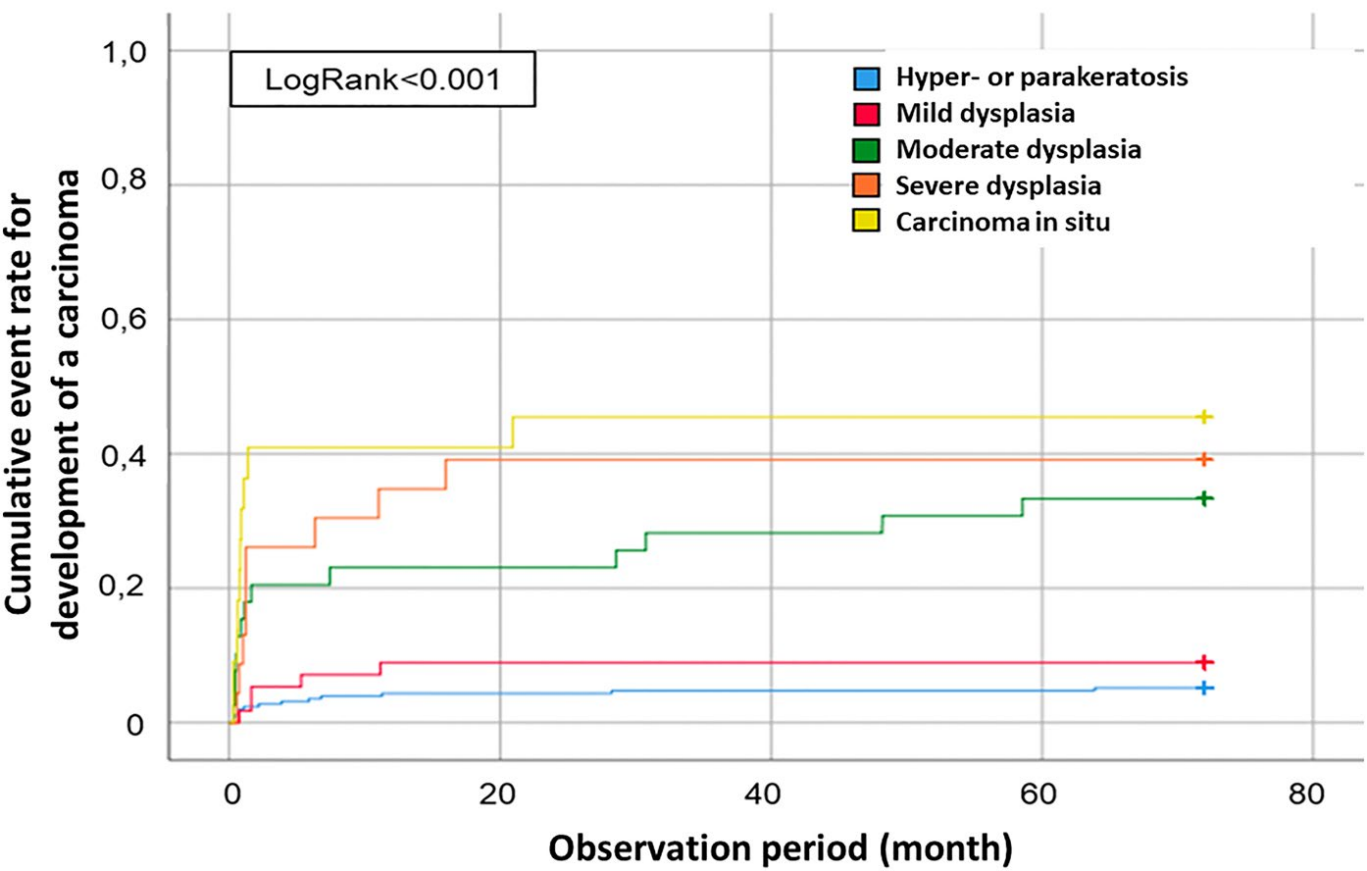
Progression to invasive carcinoma depending on the histology result of the first biopsy. Hyperkeratosis or parakeratosis and mild dysplasia show a low risk of progression to cancer, while moderate and severe dysplasia as well as carcinoma in situ have a high risk of the development of an invasive carcinoma.

**FIGURE 3:**
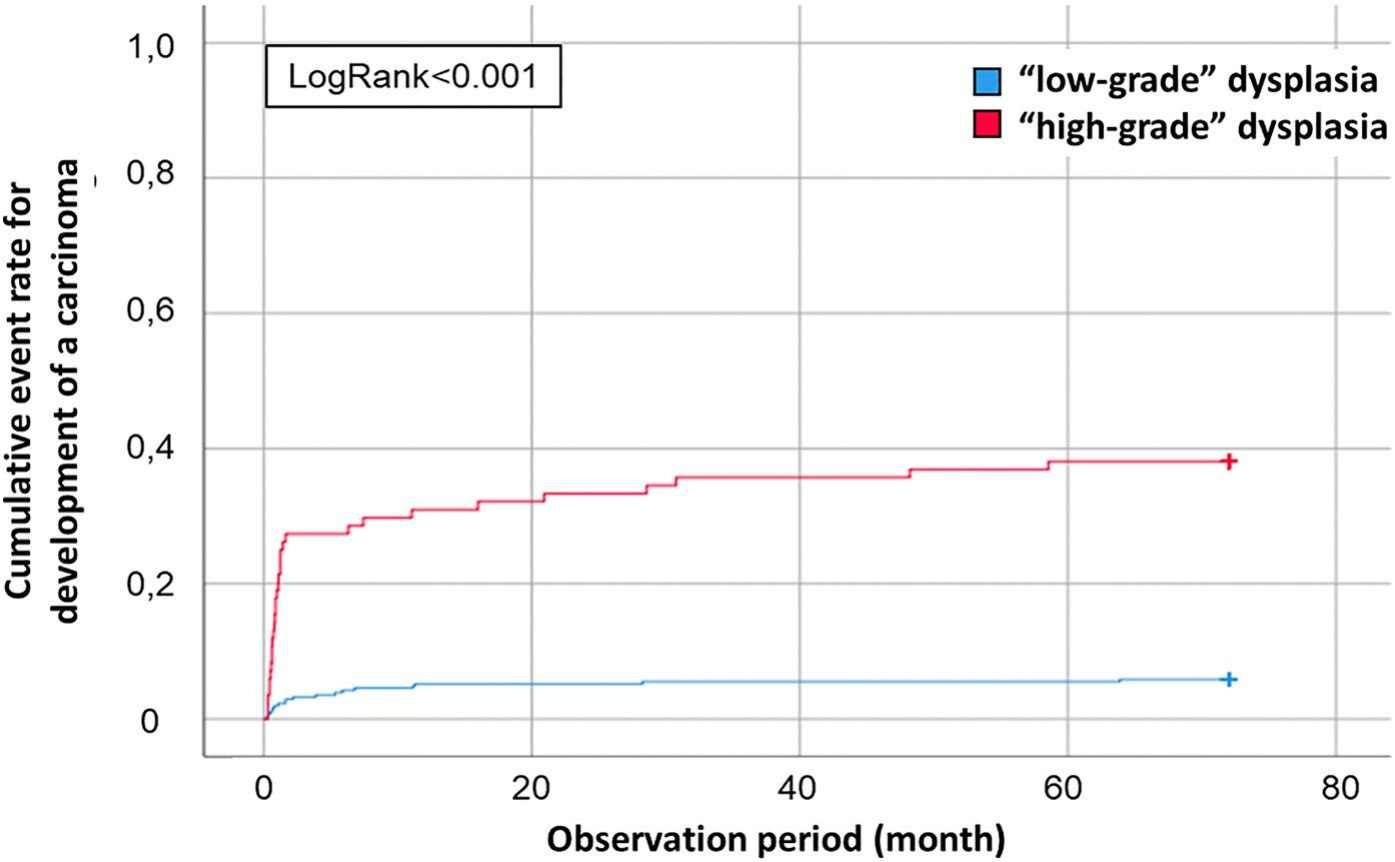
Progression to malignancy depending on the group “low‐grade” and “high‐grade” dysplasia. By pooling the results of the histology diagnoses of hyperplasia or parakeratosis and mild dysplasia in the group “low‐grade” dysplasia respectively moderate, severe dysplasia, and carcinoma in situ in the group “high‐grade” dysplasia there is a clear splitting of the curves.

Moreover, the inclusion criteria vary between studies. For example, Karatayli‐Ozgursoy et al. observed low rates of malignant precursor lesions, but the authors excluded all patients who developed a carcinoma within the first 3 months after the primary diagnosis [21]. In comparison, our results show longer mean transformation times to malignancy, but with a low median (see Table 2, Figures 2 and 3). This discrepancy might indicate a high sampling error, possibly missing the carcinoma in the first biopsy. Nevertheless, we decided not to exclude cases in which a carcinoma developed in the first 3 months. Although this would include cases in which a carcinoma was missed in the first sample, it cannot be proven with certainty that a sampling error was present in all of the cases. Excluding all cases would result in a falsely overestimated mean transformation time. As we cannot avoid errors both when excluding the cases and when including them, we have decided to show the complete data. Furthermore, the first histologic result is decisive for the clinical decision path. In our clinical routine, a re‐endoscopy with histology‐controlled resection aiming for free resection margins was indicated by the diagnosis of severe dysplasia or CIS. In the case of moderate dysplasia on histology, no routine re‐endoscopy was performed. This clinical workflow could explain the observed prolonged, yet not statistically significant, interval between the initial biopsy and cancer diagnosis: 27 months for moderate dysplasia, compared to 11 months for severe dysplasia and 15 months for CIS (see Table 2, Figures 2 and 3). However, over the entire study period, the malignant transformation rates for moderate dysplasia (41%) and severe dysplasia (44%) and CIS (55%) were comparable (see Tables 4 and S1). Thus, our data support grouping “moderate dysplasia” in the high‐grade dysplasia group of a two‐tiered system, as initially endorsed in the WHO 2017 and continued in the most recent edition [9].

WHO 2017 “low‐grade”/“high‐grade” classification system could improve histopathologic reproducibility and clinical implications [33, 34]. By recalculating our data into a two‐tier “low‐grade” and “high‐grade” classification, we found that the “low‐grade” group had a low malignancy transformation rate (6%), while the “high‐grade” group had a high malignancy transformation rate (45%) (see Table 4, Figure 3). Due to the initial pathological evaluation by the five‐tier system, recalculating into a two‐tier system is only partially feasible. In both the fourth and fifth WHO classifications, a three‐tier classification with CIS as an additional category is proposed as an alternative to the two‐tier classification. However, the fifth WHO classification prefers this three‐tier approach [14, 35]. In the present study, however, there was a clearer difference between the low‐grade and high‐grade groups than between moderate dysplasia, high‐grade dysplasia, and CIS regarding the transformation rates. In view of these data, an additional subdivision into CIS does not appear to be advantageous. One limitation of our study is that, since the two‐tier classification is only back‐calculated from the five‐tier classification, we cannot make any conclusions about the interobserver and intraobserver variability of the two systems. However, the literature shows that the new two‐tier classification cannot fully address all general histopathological challenges, such as interobserver and intraobserver variability, difficulties arising from variations in cutting plane including tangential sections, and overlaying inflammation [33, 34, 36]. One important conclusion we draw from our data is that the clinical consequence of a subsequent resection should finally be carried out for moderate dysplasia as well as for high‐grade dysplasia and CIS.

In addition, contrary to most studies in the literature, we also included leukoplakia without dysplasia in our study. In view of the 6% incidence rate of laryngeal carcinoma in our observation period, regular follow‐up investigations should also be discussed in patients with leukoplakia without dysplasia.

The study presented here examines the current state of clinical routine and is intended to provide feedback on the up‐to‐date clinical procedure. The current classifications are all based on the histopathological appearance of laryngeal lesions and do not take into account molecular and genetic changes. Initial studies are using immunohistological methods to investigate changes at the molecular level and offer the prospect that this could lead to significantly more individualized and precise conclusions. For example, the amplification of SOX2 and PIK3CA genes appears to have an influence on the malignant potential of laryngeal lesions [37, 38].

Further studies are needed to identify the most accurate molecular markers and subsequently test their significance on a larger clinical scale. Nevertheless, it is a promising prospect that specific molecular targets will find inclusion in future classifications.

## 5 Conclusion

The transformation rates of the different degrees of dysplasia vary widely in the literature. Against the background of the introduction of the binary classification by the 2017 WHO classification, in which moderate dysplasia was assigned to the high dysplasia group, we evaluated the rate of malignant transformation of the different laryngeal lesions at our center.

It was shown that moderate dysplasia had comparable rates of malignant transformation to severe dysplasia and CIS at our center. Thus, the results of our study support the classification introduced by the fourth WHO classification.

## Ethics Statement

This study was approved by the institutional ethics committee on human research of the Julius‐Maximilians‐University Würzburg (reference number 20200306 01).

## Conflicts of Interest

The authors declare no conflicts of interest.

## Supporting information


**Table S1.** Progression of vocal fold lesions from the first biopsy to the last biopsy in the observation period. From the first to the last biopsy in the observation period, the rate of progression of initially moderate dysplasia to invasive carcinoma is comparable to that of the high‐grade dysplasia and carcinoma in situ groups.

## Data Availability

All data are available on request from the corresponding author.
