# Supplementary material for: Moderate Laryngeal Dysplasia in the Context of Classification Systems and Clinical Decision Making
Source: Laryngoscope. 2025 Apr 21;135(9):3296–305. doi: 10.1002/lary.32200 (PMC12371808; doi:10.1002/lary.32200)
Supplement: Supplementary file 1 — Table S1. Progression of vocal fold lesions from the first biopsy to the last biopsy in the observation period. From the first to the last biopsy in the observation period, the rate of progression of initially moderate dysplasia to invasive carcinoma is comparable to that of the high‐grade dysplasia and carcinoma in situ groups. [file LARY-135-3296-s001.pdf]

**First biopsy**	**Latest biopsy**					
	Hyper- or Parakeratosis	Mild dysplasia	Moderate dysplasia	Severe dysplasia	Carcinoma in situ	Invasive Carcinoma
Hyper- or Parakeratosis	226 (89,7%)	4 (1,6%)	4 (1,6%)	1 (0,4%)	3 (1,2%)	14 (5,6%)
Mild dysplasia	-	47 (83,9%)	2 (3,6%)	2 (3,6%)	-	5 (8,9%)
Moderate dysplasia	-	-	22 (56,4%)	1 (2,6%)	-	16 (41%)
Severe dysplasia	-	-	-	10 (43,5%)	3 (13%)	10 (43,5%)
Carcinoma in situ	-	-	-	-	10 (45%)	12 (55%)
